# Hit That Snooze Button? Hidden Link Between Sleep Patterns, Genetic Risk, and Abdominal Aortic Aneurysm∗

**DOI:** 10.1016/j.jacadv.2024.100970

**Published:** 2024-05-08

**Authors:** Jennifer G. Co-Vu, Jeffrey P. Jacobs

**Affiliations:** University of Florida Congenital Heart Center, Gainesville, Florida USA

**Keywords:** abdominal aortic aneurysm, sleep patterns, genetic risk

**Figure:**
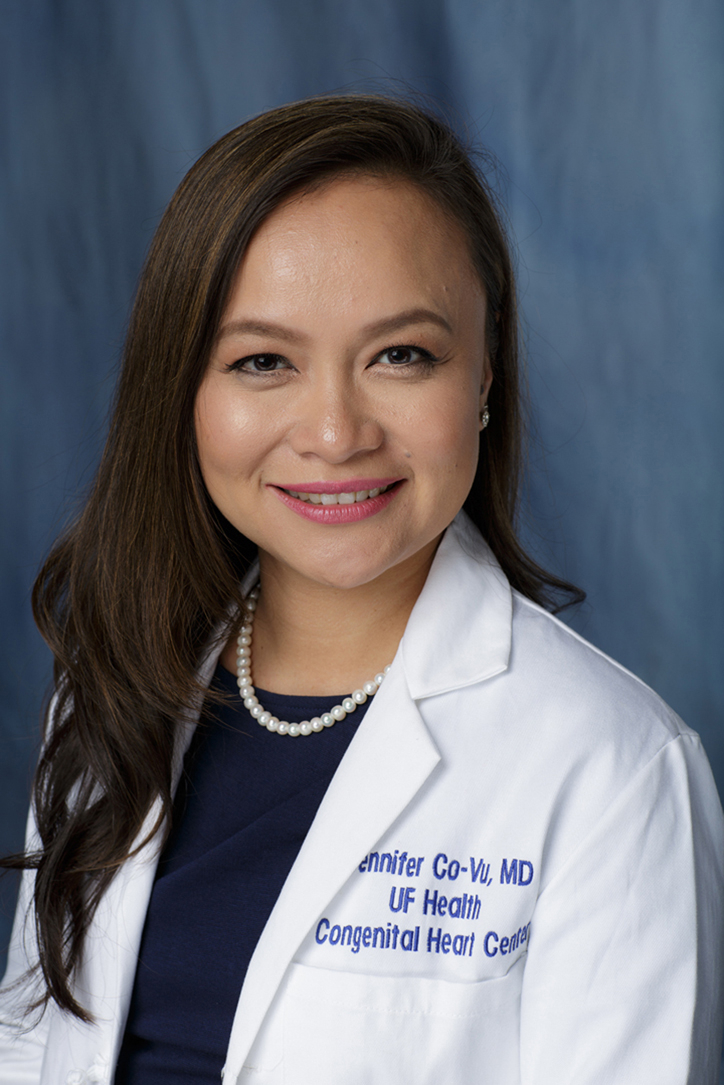

Clinical and scientific interest in the association of sleep and aortic diseases have been increasing in the recent years, and in particular, interest has increased related to the potential association of obstructive sleep apnea with the development of abdominal aortic aneurysm (AAA). Rupture of AAA has a mortality rate of up to 90%^1^, and thus, patients with AAA require careful surveillance as well as identification of factors that may contribute to the risks of AAA expansion rate.

Currently, the question regarding which factors are associated with the expansion of AAA represents an area of active ongoing investigation and has been the subject of several meta-analyses and systematic reviews.^2^, ^3^, ^4^, ^5^ Factors considered to be associated with AAA expansion include smoking, advanced age, severe cardiac disease, and a history of stroke, while diabetes mellitus, pulse pressure, body mass index, and use of statins and beta blockers appear to have no effect on the AAA expansion rate.

We congratulate Zhu et al^6^ for their thoughtful analysis titled that is published in this issue of *JACC: Advances*. The authors effectively used the UK Biobank, which is a large-scale biomedical database that recruited over 0.5 million adults aged 40 to 69 between 2006 and 2010, to investigate the association between sleep, genetic factors, and the incidence of AAA. The authors included a large cohort of 344,855 UK Biobank study participants without AAA at baseline. Sleep patterns (defined by chronotype, sleep duration, insomnia, snoring, and daytime sleepiness) were scored (range: 0 to 5), where a higher score meant a healthy sleep pattern and scores of 0 to 3 were defined as an unhealthy sleep pattern. Polygenic risk scores were used to evaluate genetic risk for AAA based on 22 single nucleotide polymorphisms. The study had an impressive follow-up (median of 12.59 years), and 1,622 AAA cases were identified. Findings show that unhealthy sleep patterns are associated with a higher risk of AAA. Study participants with poor sleep patterns and high genetic risk had a 2.5-fold higher increased risk of AAA compared to study participants with healthy sleep patterns and low genetic risk. This analysis by Zhu et al^6^ concluded: “In this large prospective study, healthy sleep patterns were associated with a lower risk of AAA among participants with low, intermediate, or high genetic risk.”

These provocative findings naturally cause the reader to ponder the question, “Does adherence to a healthy sleep pattern decrease the risk of AAA?” It is notable that a previous study from a similar cohort from the UK Biobank analyzed the relationship of healthy sleep patterns to reduced risk of cardiovascular disease, coronary artery disease, and stroke, despite genetic risk.^7^ In this elegant study by Fan et al,^7^ the authors concluded, “In this large prospective study, a healthy sleep pattern was associated with reduced risks of cardiovascular disease, coronary artery disease, and stroke among participants with low, intermediate, or high genetic risk.” The current analysis by Zhu et al^6^ makes the reader ponder whether these findings of Fan et al^7^ can be extrapolated to AAA.

The main messages of this study by Zhu et al^6^ are as follows: First, unhealthy sleep patterns, including having obstructive sleep apnea, were associated with a higher risk of AAA. Second, healthy sleep patterns, if paired with low genetic risk, were associated with lower risk of AAA. The authors have nicely discussed their findings, documenting the association of higher genetic risk increasing the risk of AAA. These findings also highlighted the importance of using this large-scale cohort of European (albeit only Caucasian) individuals in the validation of previous known hypotheses, including the association of genetic risk with AAA. It is obviously critical to explore these topics in non-Caucasian individuals as well.

Some methodological limitations of this study should be considered: The analysis depends on multiple self-reported elements of data, including all sleep behaviors as well as multiple covariates including personal history of hypertension, family history of heart disease, and lifestyle factors (eg, smoking, alcohol intake, and physical activity); these self-reported data have inherent bias that is associated with self-reporting. Furthermore, it would be interesting to readminister the questionnaire over time because sleep patterns and habits do change over time.

In summary, this thoughtful analysis by Zhu et al^6^ highlights the importance of large registries like the UK Biobank. This valuable article demonstrates the value of biomedical databases with longitudinal follow-up to facilitate scientific studies and, thus, improve global health. From a public health perspective, such analyses arising from large registries or databanks like the UK Biobank will promote healthy lifestyles such as healthy sleep patterns. Our group has similar experience collecting and analyzing multi-institutional longitudinal data about patients with hypoplastic left heart syndrome (HLHS) utilizing the National Pediatric Cardiology Quality Improvement Collaborative. We have investigated causes of HLHS mortality and promoted the standardization of innovations like the interstage home monitoring program; these data-driven innovations have significantly decreased mortality in patients with HLHS around the world. Studies like our National Pediatric Cardiology Quality Improvement Collaborative analyses and the novel study by Zhu et al^6^ clearly demonstrate the value of large biomedical databases, registries, and multicenter collaborations with diverse populations that are a true reflection of the “general population.” Such multi-institutional collaborations represent the future of the advancement of health care research in the pursuit of a healthier world.

## Funding support and author disclosures

The authors have reported that they have no relationships relevant to the contents of this paper to disclose.
